# Developmental Trajectories of Resting EEG Power: An Endophenotype of Autism Spectrum Disorder

**DOI:** 10.1371/journal.pone.0039127

**Published:** 2012-06-20

**Authors:** Adrienne L. Tierney, Laurel Gabard-Durnam, Vanessa Vogel-Farley, Helen Tager-Flusberg, Charles A. Nelson

**Affiliations:** 1 Harvard College Writing Program, Harvard University, Cambridge, Massachusetts, United States of America; 2 Department of Developmental Medicine, Children’s Hospital Boston, Boston, Massachusetts, United States of America; 3 Department of Psychology, University of California Los Angeles, Los Angeles, California, United States of America; 4 Department of Psychology, Boston University, Boston, Massachusetts, United States of America; 5 Department of Pediatrics, Harvard Medical School, Boston, Massachusetts, United States of America; French National Centre for Scientific Research, France

## Abstract

Current research suggests that autism spectrum disorder (ASD) is characterized by asynchronous neural oscillations. However, it is unclear whether changes in neural oscillations represent an index of the disorder or are shared more broadly among both affected and unaffected family members. Additionally, it remains unclear how early these differences emerge in development and whether they remain constant or change over time. In this study we examined developmental trajectories in spectral power in infants at high- or low-risk for ASD. Spectral power was extracted from resting EEG recorded over frontal regions of the scalp when infants were 6, 9, 12, 18 and 24 months of age. We used multilevel modeling to assess change over time between risk groups in the delta, theta, low alpha, high alpha, beta, and gamma frequency bands. The results indicated that across all bands, spectral power was lower in high-risk infants as compared to low-risk infants at 6-months of age. Furthermore high-risk infants showed different trajectories of change in spectral power in the subsequent developmental window indicating that not only are the patterns of change different, but that group differences are dynamic within the first two years of life. These findings remained the same after removing data from a subset of participants who displayed ASD related behaviors at 24 or 36 months. These differences in the nature of the trajectories of EEG power represent important endophenotypes of ASD.

## Introduction

Understanding the development of psychopathology has recently focused on the importance of endophenotypes. Particularly in instances where the genetic and neurological etiology of the disorder is not well characterized, endophenotypes serve as ‘intermediate phenotypes,’ which form a bridge between the biological and the psychological aspects of neuropsychiatric phenomena [1]. Endophenotypes are often biological markers associated with a given disorder and provide insight to its origins. One characteristic of endophenotypes is that they are often present in the first-degree relatives of affected individuals. Endophenotypes have been identified in family members of individuals with a variety of neuropsychiatric disorder such as depression [2], schizophrenia [3], bipolar disorder [4], and ADHD [5].

The study of endophenotypes is particularly helpful in understanding developmental disorders, such as Autism Spectrum Disorder (ASD), that are defined behaviorally, but are neurobiological in origin. In order to study endophenotypes of ASD and their relation to developmental processes, recent studies have focused on the infant siblings of children with ASD [6]–[8]. These infants are considered to be at high-risk for developing ASD given the high heritability and sibling recurrence rates of ASD [9], [10]. Despite the elevated incidence of ASD in this population (i.e. 1∶5) [6], [8], the majority (4:5) will likely not develop ASD, which makes them a key group to examine the developmental nature of endophenotypes. Some ASD endophenotypes that have been identified in high-risk infants are differences in patterns of head growth in the first year of life [11], ERP differences related to face processing in 10-month olds [12], [13], and differences in hemispheric asymmetry in alpha band EEG activity as early as 6 months of age [14]. Given that ASD is a heterogeneous disorder at both ends of the causal chain–behavioral and genetic–it is likely that there are many pathways in between that can lead to the singular ASD diagnosis and endophenotypes may help chart that intervening territory [15].

Several other candidate endophenotypes that may be functionally relevant to the etiology of ASD are related to the integration of neural networks throughout the brain. A prominent idea in the neurobiology literature is that ASD is a disorder of neural synchrony, which has its origins in the functional connections within and among regions of the brain [16]–[19]. Studies using power spectra, a measure of oscillatory amplitude that contributes to neural synchrony, have documented differences associated with ASD. Adults with ASD have higher frontal and posterior theta and posterior beta power while they also have lower frontal and posterior alpha power [20]. Children with ASD have been found to have less delta activity in frontal, central, and posterior regions and less beta activity in frontal and posterior regions [21]. Additionally, studies of event-related gamma activity have demonstrated differences in adults and children with ASD, although there are some inconsistencies in scalp location and in the direction of the differences [22]–[25]. Together these studies support the idea that neural oscillations are disrupted in ASD and EEG power captures some of the dynamics associated with this disruption.

Importantly, differences in power of resting EEG, particularly in frontal regions, have been functionally linked to cognitive functions that may be relevant to ASD. For example, variation in low alpha activity is related to individual differences in temperament [26]. Additionally, gamma power has been shown to be negatively associated with language skills and general intellectual abilities [27], while delta and theta power are inversely related to default mode network activation [28], [29]. Differences in each of these functions have also been documented in individuals with ASD [30]–[32]. Furthermore, in the case of temperament, variation therein is associated with resting EEG power in children with ASD [33]. While this appears in a broad range of cognitive and neural outcomes that are seemingly unrelated in nature, there is evidence that each one is affected in individuals with ASD [30], [32], [34]. Each metric of cognitive function may be the result of a more general set of neural process, encoded in the time-frequency domain of neural communication, for which there is evidence of disruption in ASD.

It is unclear to what extent the differences in EEG power associated with ASD are present specifically in individuals with the disorder or whether they are also present among their first-degree relatives. There is a substantial body of evidence showing that EEG power is an endophenotype of other psychopathologies such as schizophrenia [35], alcoholism [36], and depression [2], but the evidence for ASD is limited. Studies from our project on infant siblings of children with ASD have shown that properties of resting EEG activity differentiate high- and low-risk infants [37], while EEG asymmetry in alpha power is lower in high-risk infants [14]. Additionally, Elsabbagh and colleagues [38] documented higher baseline and lower induced gamma power in high-risk infants while Rojas and colleagues [39] documented no differences in baseline gamma power but higher induced gamma power of MEG activity in parents of children of ASD. Thus, despite the fact that these studies document differences in first-degree relatives of individuals with ASD, systematic study of spectral power has not been done.

Furthermore, despite the fact that changes in spectral power are evident in individuals with ASD, there are some discrepancies in the nature of the differences as they relate to the age of the study participants. For example, despite reporting similar trends in power of 3–6 Hz resting EEG, Coben et al. [21] reported lower levels of power in the 1.5–3.5 Hz range in children with ASD, while Murias et al. [20] did not report differences in this frequency range. Given that power of resting EEG changes over developmental time [40], [41], age differences of the participants may explain some discrepancies in how power varies in relation to ASD.

In the current study we examined developmental trajectories in spectral power of resting EEG in infants at high-risk for ASD. Specifically, we examined EEG activity in frontal regions of the brain, as there is structural and functional evidence that these areas are dysfunctional in ASD [42], [43] and because, as described above, frontal EEG power is associated with cognitive traits that are disrupted in individuals with ASD. We hypothesized that having an older sibling with ASD would confer risk-related differences in the levels of spectral power as well as on the rate at which they change within the first two years of life. Examining longitudinal trajectories of change, as opposed to differences at any given time point, may provide an additional metric upon which to evaluate the nature of EEG activity as an ASD endophenotype. Furthermore, given that an infant at high-risk has a range of potential outcomes [7], [44], understanding the development of their neural activity may provide insight into why an individual follows one developmental path and not another.

## Methods

### Participants

Participants were drawn from a larger sample of infants enrolled in an ongoing, longitudinal study of early development in infant siblings of children with ASD. Of the enrolled sample of 168 participants, 146 came in for a study visit, and 140 provided EEG data. From this number, 122 participants contributed useable data that are reported on in the present study. All infants had a gestational age of 36 weeks or greater, no history of prenatal or postnatal medical or neurological problems, and no known genetic disorders (e.g., fragile-X syndrome, tuberous sclerosis). Written, informed consent was provided by the parents or guardians prior to their child’s participation in the study.

Two groups of infants were included in the sample, infants at high- and low-risk for ASD. This research design builds on what we know about the high heritability of ASD [10], [30], [45] and infants are considered to be at elevated risk if they have at least one older sibling with the disorder. Indeed, based on a recent report, the prevalence of an ASD among such “high-risk” infants is approximately 20 percent (vs. 1 percent among low-risk infants) [6]. Accordingly, infants were categorized as high-risk for ASD (HRA) if they had an older sibling with a clinical ASD diagnosis (*n* = 65). A member of the study staff confirmed with the parents that the older sibling was exhibiting ASD symptomology using the Social Communication Questionnaire (SCQ) [46]. The second group of infants (*n* = 57) was recruited as a low-risk control (LRC) group. This group of infants had at least one older sibling, for whom the SCQ was used to confirm that he or she was not exhibiting ASD symptoms. None of the low-risk infants’ first-degree relatives had a known developmental disorder.

Sample demographics are presented in Table 1, displaying means for each group on characteristics of the infants and their families (note that 24 out of the 122 families provided incomplete information or declined to report it). In terms of characteristics that are thought to influence the expression of ASD–parents’ age at the infant’s birth and the infant’s birth weight–the groups were largely similar. For father’s age and birth weight, the groups were indistinguishable (*p*>0.10). However, the mothers of HRA infants were on average 1.6 years older than mothers of the LRC infants (*t*(120) = −2.15, *p* = 0.03). In terms of indices of socioeconomic status, the groups were indistinguishable on parents’ levels of education and household income (*p*>0.10; scales for these measures are listed at the bottom of Table 1).

**Table 1:** Comparison of infants in the low risk control group (LRC) and the high risk for autism group (HRA) on demographic characteristics.

	*n*	LRC	*n*	HRA	*t*(df)	*p*
Infant’s birthweight	57	7.70 lbs (1.18)	65	7.75 lbs (0.98)	−0.25(120)	0.80
Mother’s age at infant’s birth*	57	33.38 yrs (4.18)	65	35.17 yrs (4.93)	−2.15(120)	0.03
Father’s age at infant’s birth	57	36.35 yrs (5.16)	65	37.98 yrs (5.34)	−1.69(120)	0.09
Mother’s education level^a^	44	6.00 (1.61)	57	5.65 (1.79)	1.02(99)	0.31
Father’s education level^a^	44	5.68 (2.01)	57	5.55 (1.75)	0.74(99)	0.46
Household income^b^	42	7.07 (1.96)	55	7.38 (1.43)	−0.90(95)	0.36

(a) Education was reported on a scale of 1–9. (1) some high school; (2) high school graduate; (3) some college; (4) community college or 2 yr degree; (5) 4 yr college degree; (6) some graduate school; (7) master’s degree (8) doctoral degree; (9) professional degree.

(b) Income was reported on a scale of 1–8. (1) less than $15,000; (2) $15,000–25,000; (3) $25,000–35,000; (4) $35,000–45,000; (5) $45,000–55,000; (6) $55,000–65,000; (7) $65,000–75,000 (8)more than $75,000. *Significant at p<0.05.

In terms of general cognitive abilities, which were assessed at 6, 12, 18, and 24 months, both groups performed similarly (Table 2). Based on the Mullen Scales of Early Learning [47], there were no differences in the composite standard score at 6, 12, and 24 months of age, although at 18 months, the LRC infants had significantly higher scores (LRC = 107.09 [SD = 10.09]; HRA = 94.10 [SD = 15.26]; *p* = 0.02). While the groups had statistically different scores from one another at this age, it is important to note that both of these averages were well within one standard deviation (15) of the population mean (100) for this assessment.

**Table 2:** Amount of data contributed by each group at each age.

Age (mos)	*n*	Low Risk Control	*n*	High Risk Autism
6	34	47.44 (26.30)	25	39.20 (18.25)
9	32	53.70 (26.98)	30	49.82 (21.58)
12	23	46.57 (23.63)	36	43.36 (23.77)
18	11	53.82 (26.01)	24	52.25 (22.67)
24	10	60.60 (26.45)	20	48.65 (22.93)

Amount of data in seconds, standard deviation in parentheses.

### Data Collection

EEG data were collected during the 6, 9, 12, 18, and 24 month visits. The mean age of all infants at the 6-month visit was 6.33 months (SD = 0.36), at the 9-month visit was 9.15 months (SD = 0.28), at the 12-month visit was 12.22 months (SD = 0.39), at the 18-month visit was 18.22 months (SD = 0.47), and at the 24-month visit was 24.19 months (SD = 0.35).

To collect resting EEG data, infants were seated on their mothers’ laps in a dimly lit room while a research assistant engaged their attention by blowing bubbles during the entire recording session. Typically, 2 minutes of resting EEG activity were recorded. EEG data were recorded using a 64-channel Geodesic Sensor Net System or a 128 HydroCel Sensor Net System (EGI, Inc, Eugene OR). The net is comprised of an elastic tension structure forming a geodesic tessellation of the head surface that contains carbon fiber electrodes embedded in pedestal sponges. At each vertex is a sensor pedestal housing an Ag/AgCl- coated, carbon-filled plastic electrode and sponge containing saline electrolyte. Prior to fitting the sensor net over the scalp, the sponges are soaked in electrolyte solution (6cc KCl/liter distilled water) in order to facilitate electrical contact between the scalp and the electrode. In order to assure the safety and comfort of the infant, the salinity of the electrolyte solution was the same as tears.

Prior to recording, measurements of channel gains and zeros were taken to provide an accurate scaling factor for the display of waveform data. The infant’s head was measured and marked with a washable wax pencil in order to ensure accurate placement of the net, which was then placed over the scalp. Scalp impedances were checked on-line using NetStation (EGI, Inc, Eugene, OR). EEG data were collected and recorded using NetAmps 200 Amplifiers (EGI, Inc, Eugene, OR) and the NetStation software. The data were amplified, filtered (bandpass 0.1–100.0 Hz), and sampled at a frequency of 250 Hz. They were digitized with a 12-bit National Instruments Board (National Instruments Corp., Woburn MA).

At the start of the study, we employed the 64-channel Geodesic Sensor Nets, but halfway through the study, changed to the 128-channel Hydrocel Geodesic Sensor Nets because the company was no longer making or supporting the original nets. Given the timing of the implementation of the new electrode nets and the ongoing nature of this study, differing numbers of participants were tested on the 128-channel nets. The number of participants tested with each net did not differ based on risk group. However, subjects tested with the 128-channel net tended to contribute more sessions of good data (*p*<0.05). In order to ensure that the change in equipment did not influence the results of the analysis, we tested net type in our analyses but we found no statistically significant effects (*p*>0.10 in all cases).

### Data Reduction

Within NetStation, EEG data were digitally filtered (2–50 Hz) and re-referenced using an average reference that was applied after having excluded channels in close proximity to the eyes. Data were then exported to Matlab 7.6 and using EEGLAB [48] data were visually inspected; movement and electrical artifact (as evidenced by large amplitude fluctuations that exceeded +100 µV) were removed. Data segments were a minimum of 10 seconds long for inclusion in further analysis. The average segment length of usable data for LRC infants was 50.91 s (S.D. = 26.09 s) and for HRA infants was 46.39 s (S.D. = 22.35 s). As shown in Table 3, segment length increased with the age of the infants and consequently all models controlled for its effects. The number of subjects who contributed data at a given age in each group and the average amount of data they contributed is listed in Table 3. Fifty-nine percent of the sample contributed data at multiple time points. In this study, subjects contributed usable data for on average 1.96 sessions (SD  = 1.01) with a range of 1–5. The statistical methods described below accommodate both the longitudinal and cross-sectional nature of these data.

**Table 3:** Mean standard composite scores on the Mullen Scales of Early Learning MSEL for infants at low and high risk for autism.

Targeted Age	*n*	Low Risk Control	*n*	High Risk Autism
6 mos	33	94.45 (9.42)	25	96.12 (11.04)
12 mos	23	108.78 (14.11)	34	101.18 (15.37)
18 mos*	11	107.09 (10.09)	21	94.10 (15.26)
24 mos	10	108.10 (12.75)	19	107.26 (13.00)

Standard deviation in parentheses.

* Indicates significant at p<0.05.

Data were post-processed using in Matlab 7.6 wherein the *psd* function was used to calculate the power spectral density (PSD), using a 50% overlapping, 4-second Hanning window with a frequency bin of 0.25 Hz. The bands we analyzed were defined as follows: delta (2–4 Hz), theta (4–6 Hz), low alpha (6–9 Hz), high alpha (9–13 Hz), beta (13–30 Hz), and gamma (30–50 Hz). Here we report absolute PSD values, which were analyzed after being natural log transformed. This transformation is necessary for linearizing the 1/f scaling property that is characteristic of power spectra in humans [49].

The analysis reported here focused on frontal regions of the brain. One of the more consistent findings in the ASD literature is that frontal regions display differences in spectral power [20], [21], [50], [51] and activity in this area has been functionally related to behaviors that are typically disrupted in ASD [52]. The electrodes that were selected for this analysis cover an area of the scalp that is typically used in frontal EEG studies [53]. Each set of electrodes was centered on the F3and F4 electrodes of the 10–20 system, which cover the same area of the scalp (64 channel net left: 8, 9, 13, 16 and right 3, 57, 58, 62; 128 channel net left: 18, 19, 20, 23, 24, 27 and right: 3, 4, 10, 118, 123, 124). PSD were calculated for the electrodes individually and then averaged to give a mean PSD for each frequency band for the left and right frontal areas.

### Statistical Analysis

We employed multilevel modeling for change (also referred to as hierarchical linear modeling or mixed linear modeling) to assess the trajectories of change in band power. As mentioned previously, not all infants contributed viable data at each study visit, either because they did not complete the EEG portion of the study or because of technical problems with the EEG recording. However, the multilevel model for change is well suited for dealing with longitudinal data that is unbalanced [54]. We used SAS PROC MIXED and full maximum likelihood estimation for these analyses.

The mean band power values at each age in the LRC and HRA groups are listed in Table 4. In order to statistically assess the differences in these values over time and between groups, the multilevel model estimates two types of parameters that characterize a trajectory: initial status and slope. This is tantamount to regressing spectral power in a given frequency band on the main effect of age (*γ_10_*), the main effect of risk group (*γ_01_*), and the interaction between the two (*γ_11_*). We also tested a quadratic form of age (*γ_20_*), as individual growth plots suggested a curvature in the trajectories and because previous work has found quadratic effects in EEG development [40]. We included hemisphere (*γ_02_*), gender (*γ_03_*), and birthweight (*γ_04_*) as covariates, none of which interacted significantly with age. We also tested several other covariates that might influence spectral power as related to ASD including mother and father’s age at the infant’s birth. None of these variables had significant effects for any of the frequency bands and therefore were not included in the model. Thus, the hypothesized model was:
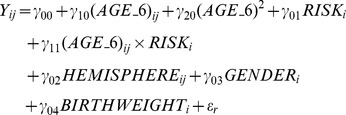
where Y*_ij_* is the level of power spectral density in each band for child *i* at time *j* and residuals ε. Parallel analyses were conducted for all frequency bands. The multilevel model for change can be specified in either a level-1 (intra-individual)/level-2 (inter-individual) format or in a mathematically equivalent composite format. Here, we used the composite format, which renders the fitted models in the form of a single linear statistical model with a complex error covariance structure. This approach permitted us to specify the error covariance structure explicitly and to account for the doubly repeated nature of the outcome, between hemispheres and across time. We used a modified compound symmetry covariance matrix that allowed the composite residuals to be homoscedastic and autocorrelated across occasions between hemispheres, with different intra-class correlations (see supporting information S1 for full specification and explanation).

**Table 4:** Mean power spectral density for each group for each frequency range at each age collapsed across hemisphere.

	Low Risk Control	High Risk Autism
Delta		
6	7.60 (0.41)	7.33 (0.47)
9	7.76 (0.43)	7.49 (0.36)
12	7.57 (0.39)	7.43 (0.44)
18	7.63 (0.34)	5.58 (0.36)
24	7.59 (0.32)	7.45 (0.33)
Theta		
6	6.69 (0.49)	6.47 (0.50)
9	6.93 (0.45)	6.61 (0.35)
12	6.97 (0.40)	6.72 (0.51)
18	6.93 (0.42)	6.88 (0.50)
24	7.02 (0.54)	6.65 (0.38)
Low Alpha		
6	5.74 (0.47)	5.53 (0.46)
9	6.02 (0.42)	5.80 (0.40)
12	6.39 (0.46)	6.06 (0.50)
18	6.25 (0.44)	6.21 (0.47)
24	6.33 (0.35)	6.08 (0.43)
High Alpha		
6	4.81 (0.49)	4.54 (0.44)
9	4.94 (0.43)	4.74 (0.36)
12	4.96 (0.42)	4.83 (0.42)
18	5.06 (0.42)	4.92 (0.31)
24	5.18 (0.27)	5.03 (0.38)
Beta		
6	4.16 (0.51)	3.81 (0.41)
9	4.29 (0.49)	4.09 (0.42)
12	4.28 (0.42)	4.07 (0.54)
18	4.17 (0.40)	4.14 (0.42)
24	4.71 (0.39)	4.13 (0.41)
Gamma		
6	3.31 (0.59)	2.97 (0.44)
9	3.45 (0.54)	3.22 (0.48)
12	3.38 (0.52)	3.13 (0.59)
18	3.14 (0.39)	3.13 (0.59)
24	3.23 (0.50)	3.03 (0.49)

Values presented in natural log of power spectral density. Standard deviation in parentheses.

## Results

**Figure 1:**
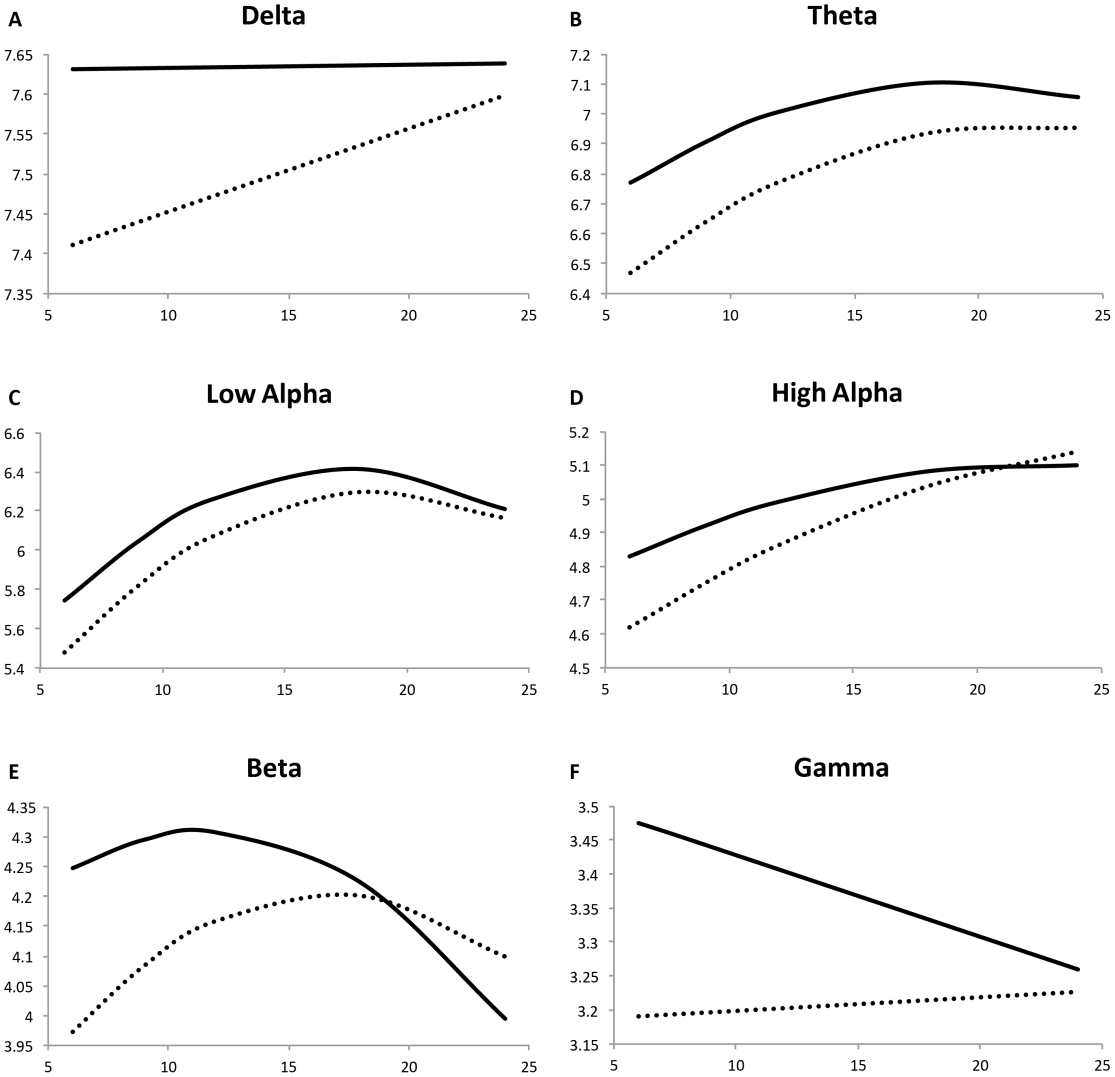
Fitted trajectories of change over time in band power (Low risk control displayed as solid line; High risk for autism displayed as dotted line). Age (in months) is on the x-axis and the natural log of spectral power (in microvolts squared) is on the y-axis.

Parameter estimates of fixed and random effects and associated significance levels are listed for all bands in Table 5 and fitted trajectories for each band are displayed in Figure 1. Differences along these trajectories (at 9, 12, 18, and 24 months) were evaluated with linear contrast tests using the Wald statistic [54].

**Table 5:** Estimates of fixed and random effects from individual growth models in which ASD risk predicts initial status and the rate of change in spectral power in infants between 6 and 24 months.

	Parameter	Delta	Theta	Low Alpha	High Alpha	Beta	Gamma
Fixed Effects							
Intercept	γ_00_	7.95***	7.25***	5.88***	5.09***	4.92***	4.34***
Age	γ_10_	0.0004	0.052***	0.116***	0.033***	0.022*	−0.012*
Age^2^	γ_20_		−0.002***	−0.005***	−0.001**	−0.002**	
ASD Risk	γ_01_	−0.220**	−0.300***	−0.262***	**−**0.212**	**−**0.274***	**−**0.285***
ASD Risk*Age	γ_11_	0.010*	0.011∼	0.012*	0.014*	0.021***	0.014∼
Hemisphere	γ_02_	**−**0.057*	**−**0.027	**−**0.003	0.037	0.131***	0.177***
Gender	γ_03_	**−**0.086	**−**0.067	0.032	**−**0.021	**−**0.129*	**−**0.209**
Birthweight	γ_04_	**−**0.033	**−**0.060∼	**−**0.021	**−**0.037	**−**0.093**	**−**0.117**
Variance Components							
	σ^2^	0.079***	0.100***	0.098***	0.076***	0.092***	0.147***
	σ_1_^2^	0.096***	0.112***	0.109***	0.099***	0.119***	0.129***
	σ_2_^2^	0.104***	0.122***	0.118***	0.098***	0.109***	0.108***

∼ p<.10; * p<.05; ** p<.01; *** p<.001.

### Delta Power

There was a main effect of group (*p* = 0.001) such that delta power was significantly lower in HRA infants at 6 months as compared to LRC infants. However, a significant group by age interaction (*p* = 0.059) indicated that the rate at which delta power subsequently changed was faster for the HRA infants. For this band, there was also no statistically significant quadratic term and thus it was eliminated from the final model. The trajectory for delta power in LRC infants had a slope that did not differ from zero (*p* = 0.929). Effectively, the LRC trajectory was flat, changing only 0.71% between 6 and 24 months, a change that was not significant as confirmed by post hoc tests (χ^2^ = 0.01, *p* = 0.929). For HRA infants, the positive slope effectively meant that delta increased by about 17%, between 6 and 24 months (χ^2^ = 9.71, *p* = 0.002). Thus, despite starting off with lower power at 6 months, the delta power trajectory for the HRA infants then converged with that of the LRC infants’ over the subsequent 18 months (Figure 1A). Post hoc tests indicated that the group differences in delta power persisted at 9 months (χ^2^ = 8.23, *p* = 0.004), and 12 months (χ^2^ = 5.85, *p* = 0.010), but by 18 and 24 months they were no longer different (χ^2^ = 1.82, *p* = 0.177 and χ^2^ = 0.11, *p* = 0.741 respectively). There was also a small, but statistically significant, hemispheric difference in delta power. The left hemisphere had about 0.75% more delta power than the right in both the LRC and HRA groups (*p* = 0.020).

### Theta Power

Theta power showed different trends than those observed for delta power. In terms of initial status at 6 months, there was a main effect of group (*p*<0.001) such that theta power was significantly lower in HRA infants. In terms of slope, LRC infants had significant linear (*p*<0.001) and quadratic (*p*<0.001) rates of change during this period. Only the linear term interacted with group with a marginal effect of this interaction (*p* = 0.066). The resultant trajectories (Figure 1B) show that, between 6 and 24 months, power increased by about 25% in the LRC infants and by 38% in the HRA infants. Due to the converging trajectories, post hoc tests indicated group differences in theta power were not significant at later time points (9 months: χ^2^ = 3.14, *p* = 0.077; 12 months: χ^2^ = 0.00, *p* = 0.952; 18 months: χ^2^ = 2.29, *p* = 0.130; 24 months: χ^2^ = 1.04, *p* = 0.309).

### Alpha Power

Alpha power was divided into low (6–9 Hz) and high (9–13 Hz) bands given previous research on the frontal alpha rhythm in infants.^53^ For low alpha, there was a main effect of group (*p* = 0.0005) such that HRA infants had lower power at 6 months of age as compared to LRC infants. The statistically significant linear (*p*<0.001) and quadratic (*p*<0.001) age terms indicated that the trajectory for the LRC infants was increasing quadratically. There was an interaction between the linear age term and group (*p* = 0.058) indicating that the HRA trajectory differed slightly in shape. This difference can be more readily visualized Figure 1C. Both the LRC and HRA trajectories demonstrated quadratic increases in low alpha power. The LRC infants increased by 37% but the faster linear rate of change in the HRA group effectively meant that low alpha power increased by 49%. In terms of the amount of power at each time point, low alpha activity remained lower in the HRA infants throughout this period with the exception of 9 months (9 months χ^2^ = 1.05, p = 0.306; 12 months (χ^2^ = 16.93, *p*<0.001; 18 months χ^2^ = 42.27, *p*<0.001; 24 months χ^2^ = 18.98 *p*<0.001).

For high alpha power, the main effect of group (*p* = 0.002) indicated that in HRA infants it was lower than the LRC infants at 6 months of age. Both linear and quadratic age terms were significant (*p* = 0.0007 and *p* = 0.006 respectively) indicating that the trajectory of change in high alpha power in LRC infants was increasing quadratically. The significant interaction between the linear age term and group (*p* = 0.015) suggested that HRA infants’ linear rate of change increased even faster than that of the LRC infants. Effectively, high alpha increased by 23% in LRC infants and by about 40% in HRA infants between 6 and 24 months of age. The result was that high alpha power in HRA infants started off lower than in LRC infants, but then appeared to cross over the LRC trajectory between 18 and 24 months (Figure 1D). Post hoc tests indicated that the amount of high alpha power was not different at 9, 12, and 18 months, but that at 24 months, the HRA infants showed more high alpha power (χ^2^ = 6.29, *p* = 0.013).

### Beta Power

For beta power, there was a main effect of group (*p* = 0.0003) indicating that beta power was lower in the HRA infants than LRC infants at 6 months. There were significant linear (*p* = 0.044) and quadratic (*p* = 0.002) rates of change in the LRC infants. Together these parameters define a beta trajectory that is curved but decreasing (Figure 1E) and results in decline of beta power of about 28% between 6 and 24 months. The interaction between group and the linear age term (*p* = 0.0007) indicates a difference in slope of the HRA beta trajectory and, as seen in Figure 1E, effectively means that beta power was changing less quickly. Across this time period, beta power declined 11% in the HRA infants. Taken together, these trajectories appear to converge and post hoc tests indicate that in terms of group differences in the amount of beta power at given time points, beta power continues to be lower in HRA infants at 9 months (χ^2^ = 4.64, *p* = 0.032), but is not longer different at 12 months (χ^2^ = 0.96, *p* = 0.327), 18 months (χ^2^ = 0.00, *p* = 0.977) or 24 months (χ^2^ = 0.20, *p* = 0.668).

In addition to these group differences, we found main effects of hemisphere, gender, and birthweight in the modeling of beta power. For both groups, the right hemisphere had more beta power than the left hemisphere (*p*<0.001). This difference translates into about 3% more beta power in the right hemisphere than the left. There were gender differences too such that boys, on average, had higher beta power than did girls (*p*<0.048). Again this difference was small; boys had about 3% more beta power than girls did. There was an effect of birth weight where infants with higher birth weight tended to have lower beta power (*p* = 0.004). There were no statistical interactions between these covariates and group nor did these covariates have effects that changed over time.

### Gamma Power

The main effect of group indicated that HRA infants had lower gamma power than LRC infants at 6 months (*p* = 0.001). The linear age term was negative and statistically significant (*p* = 0.036). There was no significant quadratic effect of age. Together, these two parameters indicate that gamma power in LRC infants was decreasing between 6 and 24 months, which resulted in a 24% decline. The interaction between the age term and group (*p* = 0.060) indicates that the trajectory of gamma power in HRA infants had a marginally different slope. Effectively, gamma power was not declining as rapidly in the HRA infants, and in fact increased by about 3.5%. Because of these different rates of change, the gamma power trajectories of these two groups appear to be converging during this time period. Despite these converging trajectories, post hoc tests indicated that the lower gamma power in HRA infants persisted through 24 months (9 months χ^2^ = 8.21, *p* = 0.044; 12 months χ^2^ = 5.17, *p* = 0.024; 18 months χ^2^ = 4.16, *p* = 0.042; and 24 months χ^2^ = 7.74, *p* = 0.006).

Similarly to beta power, there were main effects of hemisphere, gender, and birth weight in the modeling of gamma power. For both groups, the right hemisphere had more gamma power than the left hemisphere (*p*<0.001). This difference translates into about 5.5% more gamma power in the right hemisphere than the left. There were gender differences in that boys had higher gamma power than did girls (*p* = 0.004). Again this difference was small; boys had about 6% more gamma power than girls did. There was an effect of birth weight where infants with higher birth weight tended to have lower gamma power (*p* = 0.001). There were no statistical interactions between these covariates and risk status nor did these covariates have effects that changed over time.

### Sensitivity Analysis

In order to confirm that these results were not being driven by the subset of infants who developed ASD, each model was fitted after removing data from 4 participants who met ASD criteria at 24 or 36 months. The Autism Diagnostic Observation Scale (ADOS) [55], a standardized and semi-structured assessment of ASD, was scored by a trained and reliable study staff member. At the time of this study, 82 infants had been assessed on the ADOS at one or both of the time points, 4 of whom met criteria for ASD (all of whom were HRA infants). After having removed data from these 4 participants, all results remained essentially the same. The only difference was that the age by group interaction became stronger for theta (*p* = 0.037) and gamma (*p* = 0.033). Therefore, the trajectories described above remained the same confirming that these effects are characteristic of high-risk infants in general, not simply driven by the subset who go on to develop ASD symptoms.

## Discussion

### General Findings

This is the first study to explore the development of EEG in infants at high risk for ASD. The main findings are that, in comparison to infants at low-risk for ASD, infants at high risk have lower spectral power in all frequency bands at 6 months of age and that the subsequent rates of change in spectral power differ between the two groups in all frequency bands. Based on the differences in these two parameters that define the trajectories, differences in the amount of power between the two groups are dynamic across this period of development. While group differences in delta, theta, and beta power disappear by 24 months of age, power in low alpha, high alpha, and gamma bands remains different in the high-risk infants. Thus, EEG power is a promising endophenotype, but because it is age dependent, group differences at any given age, or lack thereof, may be misleading. Rather, properties of the trajectories of change in EEG power may be more robust in their capacity as intermediate phenotypes that link genetic and behavioral variation.

In addition to documenting that EEG power changes over time in these infants, we also documented that, in all cases except delta and gamma power, the shape of trajectories of change are curved. Thus, the change in these frequencies is not simply a linear shift that extends uniformly across development. The result of the differences in slope associated with risk for ASD underscores the idea that group differences in power are also dynamic. The fact that group differences in power are observed at 6 months, but not 12 or 18 months as in the case of delta, theta and beta power, that group differences disappear and then reemerge in the case of high alpha, and that group differences persist but appear to be on converging trajectories as in the case of low alpha and gamma power all foreshadow the fact that if we were to conduct this analysis later in childhood, we may observe very different patterns of relative amounts of power in each group. There are two hypotheses about what may happen. First, the patterns that we observe here are ones of normalization in which early differences in the high-risk infants disappear and later show patterns similar to those of low risk infants. Second, EEG power continues on these differential trajectories beyond 2 years of age such that group differences remain dynamic throughout development.

The patterns of early differences followed by convergence of power trajectories in delta, theta, and beta power may reflect an early maturational delay that is rectified by 24 months (or potentially shortly thereafter in the case of low alpha and gamma power). Importantly, the patterns we describe here are representative of the high-risk group and are not specific to those who develop ASD. Consequently, the convergence of power values may reflect the fact that the large majority of HRA infants (i.e. approximately 80%) will not go on to develop the disorder itself. However whether the trajectories are normative may be answered in what lies beyond the points of convergence, which will be determined by future studies that extend beyond 24 months. Many studies have documented that brain activity in siblings of individuals with ASD is different from that of the affected individual [38], [56], while there is also evidence that brain structure and function in siblings is still different from typically developing individuals [57]. Thus, reconciling whether there is normalization between high risk and typically developing infants or further differentiation along these trajectories will require further study of at risk children.

### Discussion of Band Specific Findings

Although this study is the first longitudinal analysis of resting EEG power as it relates to risk for ASD, we can evaluate the findings of the low-risk infants to previous studies of typically developing infants. In that context, the results of delta power are somewhat at odds with previous findings, while the results of low alpha power are consistent. We found that there was effectively no change between 6 and 24 months in the low risk control infants in frontal delta power, whereas Fox and Bell [53] found a decrease between 7 and 12 months of age. One potential explanation is that their delta range was defined as 1–4 and ours was 2–4 Hz. The inclusion of that extra hertz activity–which is generally of high amplitude–may have been enough to push the trend to a significant decrease. In terms of low alpha power, Fox and Bell [53] documented an increase in power of this frequency range between 7 and 12 months of age, and we not only confirm this finding, but provide evidence that the trend continues into the second year of life. Further investigation of resting frontal alpha power in the high risk group is of interest because it is correlated with joint attention [52], a cognitive function that is disrupted in ASD [58], [59] and in infants at risk for ASD [60]. Therefore, understanding the development of activity in this frequency range may have important explanatory power for cognitive functions affected in the broader ASD phenotype.

The only other longitudinal study of theta was conducted in much older children and found a decreasing amount of theta power [40]. Our results would be consistent with that finding if the quadratic rates of change continued beyond 2 years. In other words, the increasing trajectory we found may not be discrepant with the decreasing trajectory documented by Gasser and colleagues [40], rather they may represent different parts of a common quadratic trajectory.

We found that beta (13–30 Hz) power is lower in high-risk infants early on, but is no longer distinguishable from that of low risk infants after 18 months of age. In another study of infants at high risk, Elsabbagh et al. [38] found no differences in beta (20–30 Hz) in 10-month-old infants in the frontal regions. However, their baseline segments were taken from an event related paradigm in which faces were serially presented to the participants. The repetition in stimuli presentation may have had an effect that modulated the frequency content of their baselines as well.

Studies of resting gamma activity have documented that, in childhood, there are fluctuations in gamma power. For example, Takano & Ogawa documented that frontal gamma power increases between the ages of 3 and 4, but shows multiple periods of decreasing between 5 and 12 years of age [61]. Our findings that gamma power decreased between 6 and 24 months of age in low risk infants are consistent with this dynamic change over time. Elsabbagh et al. [38] found no differences in baseline gamma power in the frontal regions in 10-month-old high-risk infants. As in the case of the differences in beta power findings described previously, one possible explanation is that the baselines were affected by the serial presentation of the stimuli. Alternatively, it could be due to the structure of the baseline condition itself. Given that gamma power is sensitive to perceptual properties involved in object representation [62], a finding that appears to extend into infancy [63], one possible explanation for this discrepancy could be due the perceptual difference in the visual stimuli involved in these conditions. The participants in our study were watching bubbles, while in Elsabbagh et al. [38] they watched a fixation cross. The difference in movement and social content associated with a researcher blowing the bubbles may account for this difference, but it does not undermine the finding that the infants showed difference patterns of change in gamma power based on the genetically conferred risk for ASD.

Importantly, the changes in gamma power described here are consistent with other studies that look at the relationship between frontal gamma power in resting EEG and endophenotypes of risk for autism [39] and language impairments [27], [64]. Rojas et al. [39] found that parents of children with ASD also have lower gamma power, while Benasich and colleagues [27] found that lower gamma power in children at risk for language impairments and that this difference in correlated with language performance and IQ.

### Future Directions

Given that there is important neurocognitive development during this period [65], [66], there may be long-term consequences of these early differences in neural oscillations. The synaptic changes that characterize this period of development mean that the organization and synchrony of cortical networks is continuously changing. With synaptic pruning, myelination, and the reinforcement of synaptic connections through the period of development, the relative contribution to short range and long-range connections change over time [67]. Importantly, synchronous activity is not only the consequence of maturational and developmental processes, but helps shape such processes as well [68], thus early perturbations of neural oscillations may be disruptive of long-term cognitive outcomes. Because siblings of individuals with ASD are at higher risk not only for ASD, but also for symptoms of the broader ASD phenotype [69], disruption of neural oscillations may be related to subclinical outcomes, not just clinical outcomes.

The underlying mechanisms that account for oscillatory dynamics and the differences we document here are important topics for future research. In terms of gamma power, there is some genetic evidence that implicates inhibitory interneurons in generation and regulation of oscillations in this frequency range [70], [71]. Furthermore, interneurons have been implicated in the pathophysiology of neurodevelopmental disorders generally [72] and in ASD more specifically [73], [74]. Both computational and functional studies of gamma activity have demonstrated that interneurons are involved in the generation of neural activity within this frequency range via gap junction and GABA activation pathways [75], [76]. It is unclear whether it is through these synaptic pathways that gamma activity is affected in ASD and the broader phenotype, although some research suggests that synaptic communication is at the heart of ASD related pathophysiology [77]. Research on GABA function suggests that mutations in genes associated with GABA receptors are found in some individuals with ASD [78] and that GABA levels are abnormally high in some children with ASD [79]. Furthermore, Rubenstein and Merzenich [80] describe ASD as a disorder in the ratio of excitatory/inhibitory activity in cortical networks. Their research has identified a reduction in GABAergic signaling as a possible mechanism through which this imbalance occurs.

The extent to which these cellular mechanisms relate to endophenotypes of ASD or whether they are specific to an ASD diagnosis are issues that remain unexplored. However their resolution may provide critical information for the understanding of the biological processes that determine whether risk for ASD leads to typical or atypical outcomes. In conclusion, this paper reports that infants at elevated risk for developing autism show atypical neural oscillations across several frequency bands during the first two years of life. During this developmental window, siblings of children with ASD show markedly different developmental trajectories of change of EEG power. Characteristics of these trajectories serve as potentially important endophenotypes of ASD.

## Supporting Information

Supporting Information S1 **Error Covariance Structure.**
(DOC)
