# Supplementary material for: Developmental Trajectories of Resting EEG Power: An Endophenotype of Autism Spectrum Disorder
Source: PLoS One. 2012 Jun 20;7(6):e39127. doi: 10.1371/journal.pone.0039127 (PMC3380047; doi:10.1371/journal.pone.0039127)
Supplement: Supporting Information S1 — Error Covariance Structure. (DOC) [file pone.0039127.s001.doc]

**S1. Error Covariance Structure**

EEG data were collected both at multiple time points and from multiple regions of the brain. Thus, in the multilevel model, we specified an error covariance structure (ECS) that allowed composite residuals to be autocorrelated both over time and between brain hemispheres. We assumed that the ECS had a compound symmetric structure in which residuals are normally distributed, with zero mean vector, and are homoscedastic and autocorrelated across time within person by hemisphere. The structure was as follows:

where σ, σ_1_ and σ_2_ are the hypothesized parameters of this error covariance structure. Notice that this hypothesized structure restricts the residuals to be homoscedastic on all occasions and autocorrelated across occasions and between hemispheres as one might expect for brain data taken from the same individual's two hemispheres at different timepoints. Within hemisphere, the autocovariance is
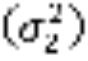
 while between occasions it is
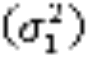
.

Compared to both the traditional compound symmetric form, which allows residuals to be autocorrelated across time but independent across hemisphere and, to the classicial error covariance structure used in repeated measures analysis of variance, the fit of the multilevel models with this hypothesized ECS was far superior. The deviance statistic associated with the full model presented herein using the modified ECS was 648.5. This is 14 points better (smaller) than with the compound symmetric form (critical value on 1 degree of freedom is 3.84) and 96.5 points better (smaller) than the classical repeated-measures form (critical value on 2 degrees of freedom is 5.99).
